# Factors Influencing Health Workers’ Acceptance of Guideline-Based Clinical Decision Support Systems for Preventive Services in Thailand: Questionnaire-Based Study

**DOI:** 10.2196/57314

**Published:** 2025-07-16

**Authors:** Tullaya Sitasuwan, Prapat Suriyaphol, Saranath Lawpoolsri, Ngamphol Soonthornworasiri, Wirichada Pan-ngum

**Affiliations:** 1 Department of Medicine, Faculty of Medicine Siriraj Hospital, Mahidol University, Bangkok, Thailand; 2 Division of Medical Bioinformatics, Department of Health Research and Development, Faculty of Medicine Siriraj Hospital, Mahidol University, Bangkok, Thailand; 3 Department of Tropical Hygiene, Faculty of Tropical Medicine, Mahidol University, 8th floor and 9th floor Tranakchit Harinasuta Building, 420/6 Ratchawithi Road, Bangkok, 10400, Thailand, 66 23069188, 66 23069188; 4 Mahidol-Oxford Tropical Medicine Research Unit, Faculty of Tropical Medicine, Mahidol University, Bangkok, Thailand

**Keywords:** clinical decision support systems, preventive health services, medical informatics, technology acceptance, continuity of patient care

## Abstract

**Background:**

A guideline-based clinical decision support system (CDSS) is a knowledge-based system designed to collect crucial data from electronic medical records to generate decision-making based on system data requirements and inputs from standard guidelines. Despite the potential to enhance health care delivery, the adoption rate of CDSSs in clinical practice remains suboptimal.

**Objective:**

This study aimed to evaluate the determinants influencing the intention to use a new CDSS in preventive care within clinical practice.

**Methods:**

A single-center, questionnaire-based, cross-sectional study was conducted among physicians and medical students responsible for providing comprehensive preventive services at the Continuity of Care Clinic, Siriraj Hospital, Thailand.

**Results:**

In total, 89 participants were enrolled. Relationships between factors impacting the adoption of CDSSs were analyzed using correlation and regression analysis. We found that physicians’ intentions to adopt the CDSS for preventive care were high, with 79% (70/89) of participants expressing their intention to use the system. According to the study’s conceptual framework, modified from the original unified theory of acceptance and use of technology model, physicians’ positive attitudes toward CDSS use in preventive services and a high level of effort expectancy emerged as crucial factors influencing the intention to use the new CDSS. The odds ratios for these factors were 5.44 (95% CI 1.62‐18.34, *P*=.006) and 7.60 (95% CI 1.55‐31.37, *P*=.01), respectively. Similar results were observed for medical students and for physicians who had graduated. The most prevalent barriers to CDSS implementation were related to physicians’ attitudes, followed by issues such as the accuracy and burden of data input, time constraints for clinicians, and the risk of workflow disruption.

**Conclusions:**

There was a high intention to adopt the CDSS in preventive care. Positive physician attitudes toward CDSS use in preventive services and effort expectancy were found to be critical factors influencing the intention to use the new CDSS.

## Introduction

### Background

In contemporary medical practice, evidence-based guidelines play a crucial role in integrating the best available evidence from various studies. These guidelines serve to support and guide physicians in making informed decisions regarding further investigation and treatment, ultimately improving the quality of care. Preventive care, among other areas, has numerous guidelines from various professional societies, recommending vaccinations and screening for diseases such as cardiometabolic diseases, cancer, and dementia. However, previous studies have revealed that clinical practice guidelines are not made full use of in real-world clinical settings [1,2]. Barriers to implementing these guidelines include a lack of a comprehensible structure and local applicability, complexity in recommended clinical actions, a lack of awareness among physicians, insufficient time for recommended preventive services, a lack of awareness among patients, and financial constraints [2-5]. To improve the implementation of standard guidelines, clinicians value a user-friendly format, clear and valid evidence, details about competency and training requirements, and guidance on how to integrate experience with evidence when tailoring recommendations to individual patients.

The advent of information technology (IT) has had a major impact on the health care sector, with 1 notable advance in clinical practice being clinical decision support systems (CDSSs). CDSSs have been defined as systems “designed to aid directly in clinical decision making, in which characteristics of individual patients are used to generate patient-specific assessments or recommendations that are then presented to clinicians for consideration” [6]. CDSSs can range in complexity, from simple alerts and reminders to diagnostic assistance, therapy planning, and prescription decision support. A guideline-based CDSS is a knowledge-based system that collects crucial data from electronic medical records (EMRs), matches these data with the system’s data requirements, and generates reasoning and decision-making based on input from established guidelines. This type of CDSS is considered a mid-level CDSS. Using a guideline-based CDSS can help to overcome obstacles associated with using traditional paper-based guidelines and enhance physicians’ adherence to recommendations [6-8].

### Literature Review

Despite increasing evidence for the benefits of guideline-based CDSSs, their adoption among outpatient physicians remains limited [9-11]. The challenges encountered during CDSS implementation stem from human factors related to system use, information quality, availability of EMR implementation, system quality, and system maintenance [11,12]. The main barriers to the implementation of mid-level CDSSs in low- and middle-income countries are inefficient CDSS-EMR system design, perception of use by physicians, and the cost of CDSS acquisition and maintenance. Additional barriers include negative beliefs about the impact of CDSSs on the clinician-patient relationship and physicians’ reluctance to adopt these systems [13-15]. To investigate the successful adoption and acceptance of CDSSs, a comprehensive understanding of the interactions between human, technological, and organizational factors is crucial. Although numerous models exist for evaluating IT acceptance and use, in this study, we used the Unified Theory of Acceptance and Use of Technology (UTAUT). The UTAUT is formulated based on 4 core determinants of intentions and usage—performance expectancy (PE), effort expectancy (EE), social influence (SI), and facilitating conditions (FC). It also includes 4 moderators of key relationships with technology—gender, age, experience, and voluntariness of use. The UTAUT model distills the critical factors and contingencies related to predicting behavioral intention to use technology, particularly technology used primarily in organizational contexts. There are a lot of theories that underlie the UTAUT model, including the diffusion of innovation theory, the theory of reasoned action, the theory of planned behavior, the motivation theory, the technology acceptance model, the personal computer utilization model, and the social cognitive theory [16].

**Figure 1.**
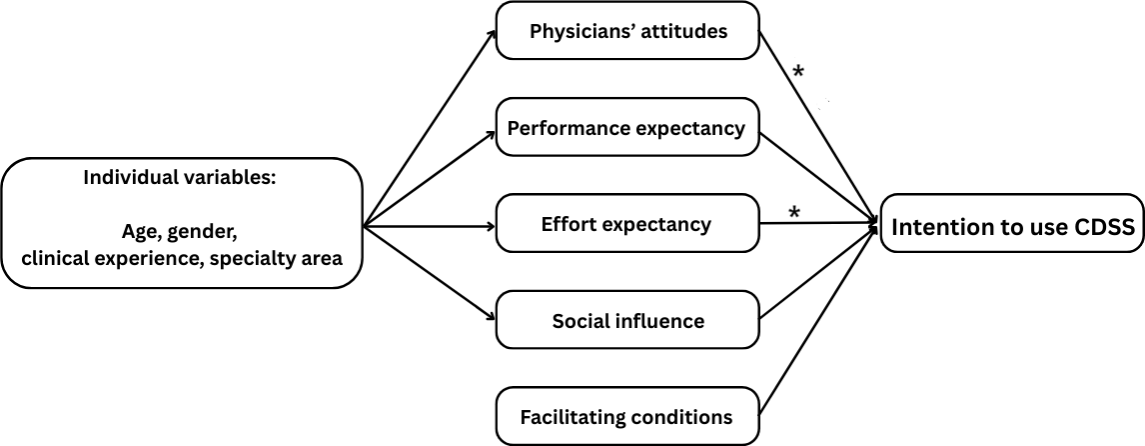
Conceptual framework used in this study * indicates a significant association at the 95% level of intention to use the CDSS. CDSS: clinical decision support system.

A meta-analysis found that the 4 primary constructs of the UTAUT model exhibit varying degrees of predictive power, with PE emerging as the strongest predictor of intention to use a new technology [17]. Previous studies have confirmed the validity of the UTAUT model in health care settings and for CDSSs, with 1 study conducted in Thailand [18-21]. Furthermore, weak positive associations have been observed between the intention to use IT and reported IT use in low- and middle-income countries, while a negative relationship has been noted between the perception of a threat to professional autonomy and the intention to adopt CDSSs [14,15]. In this study, we developed a conceptual framework based on the UTAUT model, aiming to provide a comprehensive understanding of the adoption of guideline-based CDSSs in preventive care within the health care system of an upper middle-income country. The conceptual framework encompassed two key components: (1) physicians’ attitudes toward CDSS usage, which are positively influenced by the perceived importance of preventive services, the belief that CDSSs will enhance the quality of care by improving adherence to clinical practice guidelines, and physician involvement in the CDSS development process, but negatively affected by perceived threats to professional autonomy; and (2) factors associated with CDSS PE, EE, SI, and FC, that is, the 4 factors that arise from the UTAUT model. The conceptual framework is illustrated in Figure 1.

The objective of this study was to assess user acceptance of guideline-based CDSSs in preventive care within clinical practice using the expanded UTAUT model and evaluate the readiness of medical staff and students to use this system in the Continuity of Care Clinic at Siriraj Hospital, Thailand.

## Methods

### Study Design and Participants

This study had a single-center, questionnaire-based, cross-sectional design. The participants comprised physicians and medical students who provided comprehensive preventive services at the Continuity of Care Clinic, Department of Medicine, Siriraj Hospital, between 2018 and 2021. The clinic primarily focuses on delivering ongoing care to adult patients with chronic noncommunicable diseases, aligning with the most up-to-date guidelines while promoting preventive measures such as screening for newly developed cardiometabolic diseases, cancer screening, counseling for individuals who engage in risky health behaviors, and appropriate vaccinations.

The sample size was determined based on an appropriate percentage of the finite population. The target population comprised 16 experts, 200 in-training physicians, and 300 medical students. Our primary aim was to survey a minimum of 15% of the target population, which would require a total of 75 participants to provide a sufficiently representative sample. To ensure the inclusion of individuals with valuable expertise, we planned to recruit all ambulatory medicine experts who served as permanent physicians at the Continuity of Care Clinic. This approach was intended to maximize the breadth of knowledge represented in our study, recognizing the importance of these experts’ contributions within the broader target population.

### Data Collection

Data collection was conducted using a newly developed online questionnaire, created in Google Forms, to assess user acceptance and examine the factors influencing the adoption into clinical practice of guideline-based CDSSs in preventive care. The questionnaire was pretested among a small group of physicians to ensure clarity and technical functionality. Minor revisions were made based on their feedback.

The first page of the Google Form presented participants with detailed information about the study, including its purpose, modes of contact, and data confidentiality, as well as the fact that participation was voluntary. Participants were recruited via institutional email, targeted LINE (LY Corporation; messaging app) groups, and in-person invitations. The inclusion criteria were medical students and physicians who had experience working in the Continuity of Care Clinic between 2018 and 2021 and who were currently working at Siriraj Hospital. Each participant could only respond once, and submission was allowed only after all mandatory fields had been completed. No IP addresses, email addresses, or other identifying information were collected.

### Ethical Considerations

The research protocol received approval from the Mahidol University Multi-faculty Cooperative Institutional Review Board (protocol TMEC21-052, MU-MOU CoA 608/2021). Informed consent to participate was obtained electronically by requiring participants to click “Next” before they proceeded to the questionnaire. All responses were stored securely in a password-protected institutional Google Drive account, accessible only by the research team.

### Routine Workflow for Preventive Services in the Continuity of Care Clinic at Siriraj Hospital

Outpatient services at Siriraj Hospital operate using a hybrid EMR system, in which medical records and prescriptions are initially handwritten on paper and subsequently scanned into an EMR for archival and review purposes. Laboratory results, radiological study reports, and medication prescription data are managed through separate electronic systems within the laboratory, radiology, and pharmacy departments, respectively, and are made viewable through EMR-integrated plug-ins. This system includes a basic-level CDSS, which provides pop-up reminders for drug allergies, renal function, and high-alert medications such as warfarin. A CDSS that retrieves data directly from the EMR is not applicable in this system.

At the Continuity of Care Clinic, the primary focus is on maintaining continuity of care for patients with chronic noncommunicable diseases, while simultaneously promoting preventive services. Due to the limitations of the EMR system, paper-based guidance for preventive care is incorporated into clinical workflows. This approach aims to ensure the consistent delivery of preventive services to each patient, despite the rotation of different physicians.

### Online Questionnaire

The questionnaire consisted of 22 items, including questions regarding demographic details and items related to physicians’ attitudes, EE, PE, SI, and FC, formulated in accordance with the structured questions of the UTAUT model [16]. Questions pertaining to physicians’ attitudes were derived from factors identified in previous studies as being influential on usage intentions [14,15,22]. Responses were recorded on a 5-point Likert scale ranging from “strongly agree” to “strongly disagree.” Among these constructs, physicians’ attitudes and EE had 1 item that was negatively worded. The questionnaire was initially developed in English and subsequently translated into Thai using the translation-back-translation method. Before completing the questionnaire, participants were asked to view a demonstration video outlining the functionality and use of the proposed CDSS.

### Introductory Video for the Proposed CDSS

The study participants were introduced to the proposed CDSS through an informative online video. The video was expertly created by an ambulatory medicine specialist who possessed extensive expertise in integrating preventive care into clinical practice and was also highly familiar with the EMR system employed at the study site. This video provided participants with invaluable insights into the prevailing challenges within the workflow and offered a firsthand experience of the system’s performance. The demonstration included a comprehensive overview of data entry methods, advanced data visualization capabilities, and personalized recommendations for preventive services. In addition, the video covered essential information concerning support services and regular maintenance of the clinical rules associated with these systems. A summary of the video narration and selected snapshots from the demonstration video are provided in Multimedia Appendix 1.

### Statistical Analysis

Data were analyzed using the R programming language (R Core Team). The descriptive statistics are presented as mean, median, SD, minimum, and maximum. Logistic regression analysis was used to identify factors associated with the acceptance of guideline-based CDSSs in preventive care. The final model was selected through the application of forward and backward selection methods in multiple logistic regression analysis. The statistical significance of the final model was evaluated using the Wald test.

## Results

### Demographic Data

A total of 89 participants took part in this study. Their baseline characteristics are presented in Table 1. Among the participants, 45 (51%) were male. The median age of participants was 25 (range 22-64) years. The majority of participants (49/89, 55%) were medical students, with 28 participants (32%) in their fifth year and 21 participants (24%) in their sixth year. Furthermore, 40 participants (45%) were medical graduates with varying levels of experience; 4 (5%) were general practitioners, 11 (12%) had undergone training in an internal medicine residency program, and 25 (28%) were internal medicine specialists, including 16 individuals (18%) who specialized in ambulatory medicine. The medical graduate participants had clinical experience ranging from 2 to 40 (median 8) years.

**Table 1.** Demographic data of study participants.

Characteristic	Statistical value (N=89)
Age (years), median (range)	25 (22‐64)
Sex (male), n (%)	45 (51)
Clinical experience, n (%)	
Medical student	49 (55)
Fifth year	28 (32)
Sixth year	21 (24)
Graduated physician	40 (45)
General practitioner	4 (5)
Internal medicine resident	11 (12)
Internal medicine specialist	9 (10)
Ambulatory medicine expert	16 (18)
Duration of medical doctors’ professional practice (years), median (range)	8 (2-40)

### User Acceptance Measures

After viewing the demonstration video of the proposed system, 70 participants (79%) expressed a definitive intention to use the system if it were available. However, 18 participants expressed reasonable concerns about its usage, while 1 participant refused outright to use the new system. Notably, there were no differences in the intention to use the proposed system based on the participants’ baseline characteristics. Nevertheless, within the medical student group, it was observed that sixth-year medical students exhibited a higher comfort level with adopting the new system compared with their fifth-year counterparts, with acceptance rates of 95% and 68%, respectively.

The mean (SD) and median (range) scores for each question are presented in Table 2. The scoring system for each question had a maximum value of 5. For negative questions, appropriate score conversions were implemented before calculating the total score for each construct.

**Table 2.** Scoring of each question assessing user acceptance.

Determinants of intentions and usage	Full score^a^	Mean (SD)	Median (range)
PA^b^			
Perceived importance of disease prevention	5	4.89 (0.34)	5 (3-5)
Perceived improved quality of care by increased adherence to clinical practice guidelines	5	4.708 (0.53)	5 (3-5)
Perceived threat to professional autonomy^c^	5	4 (1)	4 (1-5)
Involvement in the CDSS^d^ development process	5	4.17 (0.84)	4 (2-5)
Total PA score	20	17.78 (1.74)	18 (12‐20)
PE^e^			
Perceived usefulness	5	4.64 (0.59)	5 (3-5)
Job-fit	5	4.67 (0.54)	5 (3-5)
Outcome expectations	5	4.55 (0.74)	5 (2-5)
Total PE score	15	13.87 (1.60)	15(9-15)
EE^f^			
Ease of use	5	4.29 (0.84)	4 (1-5)
Complexity^c^	5	3.75 (1.20)	4 (1-5)
Total EE score	10	8.05 (1.78)	8 (2-10)
SI^g^			
Subjective norm	5	4.19 (0.75)	4 (3-5)
Social factors	5	4.11 (0.92)	4 (1-5)
Image	5	3.76 (1.16)	4 (1-5)
Total SI score	15	12.07 (2.18)	12 (5‐15)
FC^h^			
Perceived behavioral control	5	4.40 (0.75)	4 (2-5)
Facilitating conditions	5	4.26 (0.76)	4 (2-5)
Compatibility	5	4.25 (0.88)	4 (2-5)
Total FC score	15	12.91 (1.98)	13 (6‐15)
Total score	75	64.67 (7.25)	65 (42‐75)

a Measurement scale: 1=strongly disagree, 2=disagree, 3=neutral, 4=agree, 5=strongly agree.

b PA: physician’s attitude.

c Indicates a negative question; a higher score means less concern about these items.

d CDSS: clinical decision support system.

e PE: performance expectancy.

f EE: effort expectancy.

g SI: social influence.

h FC: facilitating conditions.

The correlation between each construct and the intention to use the proposed system is presented in Table 3. A significant positive correlation was found between the physicians’ attitudes, PE, EE, and their intention to use the system.

**Table 3.** The correlation matrix between user acceptance measurement and intention to use the new clinical decision support system.

Variable	PA^a^	PE^b^	EE^c^	SI^d^	FC^e^	IU^f^
PA						
Spearman rank	1	0.58	0.45	0.43	0.48	0.40
*P* value	—^g^	<.001	<.001	<.001	<.001	<.001
PE						
Spearman rank	0.58	1	0.62	0.51	0.67	0.29
*P* value	<.001	—	<.001	<.001	<.001	0.01
EE						
Spearman rank	0.45	0.62	1	0.25	0.40	0.44
*P* value	<.001	<.001	—	0.02	<.001	<.001
SI						
Spearman rank	0.43	0.51	0.25	1	0.68	–0.04
*P* value	<.001	<.001	0.02	—	<.001	0.69
FC						
Spearman rank	0.48	0.67	0.40	0.68	1	0.14
*P* value	<.001	<.001	<.001	<.001	—	0.20
IU						
Spearman rank	0.40	0.29	0.44	–0.04	0.14	1
*P* value	<.001	0.01	<.001	0.69	0.20	—

a PA: physician’s attitude.

b PE: performance expectancy.

c EE: effort expectancy.

d SI: social influence.

e FC: facilitating conditions.

f IU: intention to use the clinical decision support system.

g Not applicable.

### Factors Associated With Intention to Use the CDSS

The outcome from the logistic regression analysis is presented in Table 4. Physicians’ positive attitudes toward preventive medicine and the integration of new technology into clinical practices were found to be significant factors associated with the adoption and use of the new CDSS, with adjusted odds ratios (OR) of 8.41 (95% CI 2.54‐27.82). The EE was also associated with the intention to adopt the CDSS (adjusted OR 11.71, 95% CI 2.45‐55.95). However, PE, SI, and FC did not reach statistical significance in their association with the adoption of the proposed system. In addition, individual factors, such as age, sex, clinical experience, and duration of clinical practice, did not appear to influence the likelihood of adopting the proposed CDSS.

**Table 4.** Logistic regression results.

Determinants of intentions and usage	Crude OR^a^ (95% CI)	*P* value	Adjusted OR^b^ (95% CI)	Final model OR (95% CI)	*P* value
Physician’s attitude^c^	8.09 (2.55‐25.63)^d^	<.001	8.41 (2.54‐27.82)^d^	5.44 (1.62-18.34)^e^	.006
Performance expectancy^c^	2.78 (0.98‐7.85)	.05	2.78 (0.94‐8.26)	—^f^	—
Effort expectancy^c^	11.33 (2.43-52.85)^e^	.002	11.71 (2.45-55.95)^e^	7.60 (1.55-31.37)^e^	.01
Social influence^c^	0.97 (0.35‐2.72)	.96	0.84 (0.29‐2.43)	—	—
Facilitating conditions^c^	1.73 (0.62‐4.82)	.30	1.63 (0.57‐4.66)	—	—
Age	0.96 (0.91‐1.01)	.12	—	—	—
Sex^g^	1.11 (0.40‐3.07)	.84	—	—	—
Clinical experience					
Medical student	1	—	—	—	—
IM^h^ resident and internist	0.93 (0.26-3.39)	.91	—	—	—
Ambulatory medicine expert	0.51 (0.15-1.81)	.30	—	—	—
Duration of clinical practice					
0‐3 years	1	—	—	—	—
4‐10 years	0.97 (0.27‐3.50)	.96	—	—	—
>10 years	0.41 (0.11‐1.49)	.18	—	—	—

a OR: odds ratio.

b Adjusted by age and clinical experience group because the result from the univariable analysis showed a *P* value <.20.

c Where values lower than the mean of the total score were treated as baseline, that is, odds ratio=1.

d *P*<.001.

e *P*<.05.

f Not applicable.

g Where being male was treated as the baseline, that is, odds ratio=1.

h IM: internal medicine.

The final model consensus was established through the implementation of the forward selection and backward selection methods, using the Wald test. The integrated final model encompassed all determinants associated with the intention to adopt the CDSS, along with the demographic variables indicated in Table 4. The factors influencing the adoption and use of the proposed CDSS were identified as the physicians’ attitudes (odds ratio 5.44, 95% CI 1.62‐18.34, *P*=.006) and EE (OR 7.60, 95% CI 1.55‐31.37, *P*=.01), visualized in the study’s conceptual framework in Figure 1.

Subgroup analysis demonstrated that there were no significant differences in the logistic regression results between medical students and graduated physicians, as indicated in Multimedia Appendix 2. Nevertheless, it is important to highlight the notable factors associated with the intention to adopt the CDSS, which were identified as being physicians’ attitudes and EE.

### Benefits of and Barriers to CDSS Adoption in Clinical Practice

All study participants agreed that the proposed CDSS would bring benefits to their clinical practice. Specifically, 84 participants believed that the proposed system encompassed multiple components that would prove advantageous in their clinical practices. A summary of the participants’ preferences and barriers to their adopting the CDSS is provided in Table 5.

**Table 5.** The benefits and barriers of the proposed clinical decision support system.

Benefits and barriers	Frequency, n (%)	
Benefits of CDSS^a^		
Data gathering and data visualization	79 (89)	
Provides supplementary information for recommended disease screening	70 (79)	
Provides additional information regarding recommended vaccines	68 (76)	
Individualized suggestions for further preventive services	61 (69)	
Integration with the doctor’s order sheet	50 (56)	
Information to improve clinical decision-making	49 (55)	
Provides flexibility in use, not mandatory for every patient	37 (42)	
Barriers to CDSS adoption		
Other physicians usually take care of prevention services	6 (7)	
The use of the CDSS would require them to allocate more time to each patient	5 (6)	
Potential disruption of the current workflow with the implementation of the CDSS	4 (5)	
The burden of data input outweighs the benefit of the CDSS	3 (3)	
Leakage of patients’ personal data	3 (3)	
The CDSS will not improve my performance	2 (2)	
The accuracy of data input	2 (2)	

a CDSS: clinical decision support system.

The majority of participants (70/89, 79%) reported having no concerns about implementing the new system in their clinical practice, while 19 participants raised concerns regarding the proposed CDSS. Among them, 4 participants had multiple concerns, while the rest expressed a single specific concern about the new system. The most prevalent barrier identified for the adoption of the new system was the participants’ perception that they do not play a primary role in preventive services, which subsequently led to their decision not to use it.

## Discussion

### Principal Results and Comparison With Previous Work

This study suggests that a majority of medical professionals, including students and graduates, have a favorable attitude toward and intention to use a CDSS in their clinical practice. Factors, such as physicians’ attitudes toward the use of CDSSs in preventive services and its perceived ease of use, play an important role in the adoption and use of the proposed system. The primary barrier to CDSS adoption arises from physician-related factors. If physicians are not actively involved in preventive services or do not prioritize them, they would be less likely to employ the proposed CDSS.

This study was undertaken to evaluate the acceptance of a new CDSS in preventive services. It was postulated that physicians differ from other users of IT due to the impact on their professional work of implementing an automated system that influences the comprehensiveness of their clinical management and adherence to clinical practice guidelines. However, physicians may perceive this disruption as a threat to their professionalism [23].

Our model indicates that a physician’s attitude was one of the most influential factors determining their intention to use the new system. From the mean scores of each question included in this construct, we found that the highest score was for physicians recognizing the importance of disease prevention, which aligns with the primary function of the CDSS. The perception that the system can enhance the quality of health care delivery by promoting adherence to clinical practice guidelines ranked second. This finding is consistent with previous research emphasizing that physicians’ decision to use IT relies on the usefulness and compatibility of any new technology with their clinical practice [24]. Physicians are more inclined to accept a system that they think will improve their performance. However, the perceived threat to professional autonomy, which has been identified as a significant deterrent to CDSS adoption among physicians [15,23], received the lowest mean score and exhibited the widest range of scores in this construct. This suggests that participants had different concerns regarding the threat to their professional autonomy posed by the proposed CDSS. Nonetheless, most participants paid little attention to this factor, as evidenced by the median score of 4 out of 5. The level of clinical decision support provided by the CDSS can explain this discrepancy. In our study, the CDSS is intended to serve as a supportive tool for physicians’ decision-making according to well-established clinical practice guidelines. The function of the proposed CDSS primarily involves clinical data gathering from EMR, data visualization, and recommendations for routine preventive services such as cancer screening and age-appropriate vaccination. These tasks do not rely heavily on individual clinical expertise, and the system allows physicians to make their own choices based on their experience and doctor-patient discussions. The low-to-medium level of clinical decision support provided by the CDSS may account for the limited concern expressed regarding the threat to professional autonomy in this study.

EE emerged as another influential factor determining the intention to use the new system. This finding is consistent with previous studies [15,20,21] and aligns with measures in the DeLone and McLean [25] information system success model that suggest ease of use is related to user satisfaction and adoption. The end user interface arrangement, which should be simple, user-friendly, and matched with a physician’s thinking process, also constitutes a crucial factor contributing to system adoption [26].

The highest positive response on PE (mean score of 13.87 out of 15; 93%) reflected that participants expressed a strong belief in the system’s potential usefulness. This is likely influenced by the demonstration video about the function and use of the proposed CDSS, which was made by an experienced clinician involved in preventive services at the study site to highlight how the system could address existing workflow gaps. Furthermore, the proposed CDSS has the potential to enhance physicians’ performance, improve their effectiveness, and reduce the time spent on preventive services during patient encounters. The observed marginal positive relationship between PE and the intention to use the system (*P*=.05) was consistent with the expectation that perceived usefulness is a key determinant of adoption intention.

SI was not found to significantly affect the intention to use the CDSS in our study. This finding is consistent with previous literature suggesting that physicians differ from other groups of IT users, as they tend to prioritize a system’s perceived usefulness over ease of use or peer influence within their organization [23]. However, this result may be attributable to the preimplementation context of our study, in which participants had no previous experience using the system and limited exposure to peer usage or established institutional norms surrounding its adoption.

Previous studies have indicated that SI may exert a greater effect, particularly among medical students, once a system has been actively implemented and its use becomes more visible within the learning or clinical environment [27]. Although our study was conducted during medical students’ clinical clerkship phase, when decisions are likely shaped by clinical supervisors and perceived task relevance, peer and faculty endorsement may become increasingly influential as the CDSS becomes embedded in routine clinical workflows. Further research following system implementation is warranted to better understand the evolving role of social dynamics in CDSS adoption.

Subgroup analysis revealed that the factors influencing the intention to adopt the new CDSS did not differ significantly between medical students and physicians who had graduated. Correlation analysis indicated a significant positive correlation between physician’s attitude, PE, EE, and intention to use the CDSS, consistent with several previous studies [15,20,21,28].

The rate of intention to adopt the proposed CDSS in this study was remarkably high (79%). No statistically significant differences were observed in the baseline characteristics of physicians related to the intention to adopt. However, there was a disparity in intention to adopt rates within the medical student group. Sixth-year medical students exhibited a higher comfort level with the new system compared with fifth-year medical students, with acceptance rates of 95% versus 68% (*P*=.04). These findings warrant further discussion. During medical education in Thailand, fifth-year medical students have had limited clinical exposure (less than 2 years) and are under the continuous supervision of medical staff. Consequently, they may not consider clinical judgment to be an integral part of their responsibilities. In contrast, sixth-year medical students engage in clinical clerkship, which distinguishes them from fifth-year medical students. Their mindset may differ, resulting in contrasting attitudes toward the benefits and usefulness of technology in their job, which could in turn influence their adoption rates. Furthermore, greater clinical exposure enables sixth-year medical students to recognize the substantial cognitive burden of being a general practitioner. They may perceive the system as being more valuable in alleviating their cognitive load than fifth-year medical students.

Qualitative analysis of free-text comments revealed that physicians regarded the system’s ability to gather data from various sources in EMR and present these data in a recognizable and comprehensible format as the most beneficial aspect of the proposed CDSS. In addition, the system provides patient counseling information on preventive services, which was highly valued. These results align with those of previous studies, in that physicians perceive the benefit of CDSSs mainly in terms of accessibility to standardized information and the facilitation of patient discussion [19,21]. Automatic data gathering and visualization have also frequently been cited as favorable aspects of a CDSS [29]. These findings suggest that preventive services have relatively low priority in current clinical practice, primarily due to challenges in acquiring individual patient data and obtaining comprehensive information for patient counseling. A CDSS can effectively bridge this gap.

Regarding system performance, the results indicated that more than half of the study participants would prefer a single integrated system that incorporates a patient’s history, laboratory results, vaccination records, and a linked appointment system. However, some physicians favored the use of the CDSS as an optional tool for each patient visit. This finding should be taken into account during system design. An “add-on” system is perceived as being advantageous as it allows physicians to exercise their own choice. Nonetheless, this feature should be considered carefully, as it could pose a threat to system implementation due to low system usage [30].

Most study participants reported that they would use the proposed CDSS without any concerns. However, some participants expressed apprehension about adopting the CDSS. Regarding physician-related factors, physicians’ attitudes emerged as the primary barrier. As preventive services are not obligatory for all physicians, those who are not involved in such tasks or do not prioritize health promotion may be reluctant to use the proposed CDSS, as it could increase the time required for each patient visit and disrupt their routine workflow.

Regarding patient-related factors, the respondents expressed concerns about the accuracy of data input, as the system allows manual input of health maintenance data obtained elsewhere. Consequently, unverified data may lead to incorrect data being entered, which could subsequently impact the recommendations made during the use of the CDSS. This concern has been identified in previous studies with regard to information quality [31,32].

Finally, system-related factors were also identified as barriers to adoption. The burden of data input was the primary concern from this perspective. In addition, time constraints for clinicians and the potential disruption to workflow were considered important system design factors. These findings align with previous qualitative studies suggesting that a failure to balance the data input burden, the presentation of clinically relevant information, and integration into the workflow can negatively impact the adoption of CDSSs [29,30,33].

### Study Implications

This study suggests that a carefully designed CDSS, featuring a simple, user-friendly interface, would be readily adopted by physicians. A potential application of this study is the development and implementation of the proposed CDSS described in the demonstration video. For successful implementation, several key factors should be considered, such as ensuring interoperability with existing EMR systems, developing a user-friendly interface, and integrating standardized security protocols. Streamlining data input and involving patients, while allowing physicians to verify and adjust data, may reduce the documentation burden.

Beyond system design, physician attitudes play a critical role in the adoption and use of a CDSS in the long term. Therefore, it is imperative to emphasize the incorporation of preventive services into the holistic care model during medical education, particularly in the context of chronic disease follow-up in ambulatory settings. In addition, long-term use of a CDSS requires institutional support, including ongoing training and effective change management strategies to facilitate integration into clinical practice.

### Future Work

While this study demonstrates there would be a high rate of adoption of the proposed CDSS, some concerns remain among physicians, particularly regarding potential disruptions to clinical workflow, increased time spent per patient, and the burden of data input. Future qualitative studies will be important to guide the development of an improved system and to inform strategies for effective organizational implementation. In addition, addressing concerns regarding autonomy and emphasizing the role of CDSSs in supporting clinical decision-making may further encourage adoption. Long-term multicenter studies evaluating the system’s impact on clinical outcomes, as well as the perceptions of physicians and patients during clinical visits, would be valuable. In particular, these studies should explore how the use of a CDSS to guide clinical decision-making affects physician attitudes, patient trust, and the overall quality of the clinical encounter.

### Strengths and Limitations

This study had several strengths. It represents the first evaluation of the intention of physicians to adopt a guideline-based CDSS in Thailand. A questionnaire was administered, incorporating determinants from the UTAUT model and factors identified to be salient in Southeast Asia, including physician attitudes toward new technology. Furthermore, a unique video was used to introduce the proposed system, featuring an experienced clinician demonstrating preventive services and addressing current workflow challenges. The video showcased the end user interface, data entry methods, CDSS performance, and integration with the existing EMR system, providing participants with an experience to aid their decision-making process.

However, the study also had some limitations. First, it was conducted at a single center, Thailand’s largest medical school hospital, potentially limiting the generalizability of our findings to other types of hospitals with differing EMR systems and limited IT support. Second, the inclusion of participants familiar with the continuity of care model incorporating preventive services resulted in a skewed-left distribution and wide CIs in the conceptual framework and logistic regression analysis.

Furthermore, this cross-sectional study examined preadopters of the CDSS, with self-reported constructs based on the demonstration video, impeding the determination of causal relationships. It is crucial to acknowledge potential differences in attitudes between preadopters and postadopters, limiting the generalizability of the study’s results to the early stages of CDSS implementation.

### Challenges During the Research

This study was conducted during the COVID-19 pandemic, which posed several challenges. Hospital services for nonemergency cases were reduced by approximately 50%, and medical students transitioned to online learning. As a result, it was difficult to recruit study participants, particularly medical students, as they were not physically present at the clinic and were dispersed across various off-campus locations. Similarly, many graduated physicians were reassigned from the Continuity of Care Clinic to COVID-19 patient care duties. Furthermore, the closure of outpatient services greatly limited clinical exposure for medical students. Consequently, only approximately half of the fifth-year medical students had the opportunity to gain experience in providing comprehensive preventive services at the Continuity of Care Clinic during the study period.

### Conclusions

The use of CDSS in clinical management has the potential to enhance the quality of health care delivery. This study reveals a noteworthy inclination among physicians to embrace a guideline-based CDSS specifically tailored for preventive care. Furthermore, there were no significant differences in the intention to adopt the system based on diverse baseline characteristics of physicians.

Drawing upon the study’s comprehensive conceptual framework, it was evident that positive physician attitudes toward incorporating CDSSs in preventive services, along with their perceived ease of use, emerged as the most pivotal determinants influencing the intention to use the novel CDSS. Conversely, factors such as PE, SI, and FC had minimal influence over the intention to adopt the proposed system. This study identified a range of common obstacles to the adoption of a CDSS, including physicians’ attitudes, data input accuracy and burden, physicians’ time constraints, and the potential disruption to existing workflow patterns.

## Supplementary material

10.2196/57314 Multimedia Appendix 1 Supplementary material.

10.2196/57314 Multimedia Appendix 2 Logistic regression of the subgroup analysis.

## Abbreviations

CDSS: clinical decision support system
EE: effort expectancy
EMR: electronic medical record
FC: facilitating conditions
IT: information technology
OR: odds ratio
PE: performance expectancy
SI: social influence
UTAUT: Unified Theory of Acceptance and Use of Technology
