# Supplementary material for: Factors Influencing Health Workers’ Acceptance of Guideline-Based Clinical Decision Support Systems for Preventive Services in Thailand: Questionnaire-Based Study
Source: JMIR Hum Factors. 2025 Jul 16;12:e57314. doi: 10.2196/57314 (PMC12286562; doi:10.2196/57314)
Supplement: Multimedia Appendix 1 [file humanfactors-v12-e57314-s001.docx]

**Supplementary Materials**

**Factors Influencing Health Workers’ Acceptance of Guideline-based Clinical Decision Support Systems (CDSS) for Preventive Services in Thailand: A Questionnaire-based Study**

A summary of the narration from the demonstration video of the proposed CDSS.

**Part 1**The video introduces the proposed clinical decision support system (CDSS), which is designed to support preventive care and disease screening for patients receiving continuous care at the Continuity of Care Clinic. This system is intended to replace the traditional paper-based Adult Health Maintenance Checklist*.* The video addresses current workflow challenges, such as retrieving data from multiple sources. These sources include laboratory results, medical records, procedure notes (e.g., colonoscopy), and radiology reports. The workflow challenges are anticipated to be resolved through the user-friendly digital interface of the CDSS, which integrates seamlessly with the hospital’s electronic medical records (EMR) system.


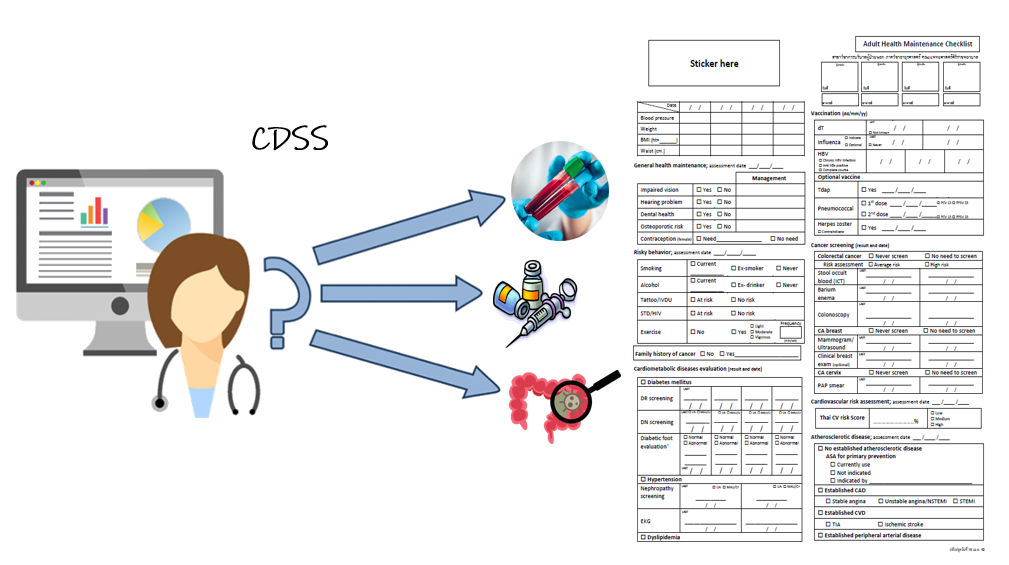


**Figure S1.** First snapshot from the demonstration video

**Part 2**The new system, the **Si**riraj **C**linical **D**ecision support system in **P**reventive services (SiCDP), enables rapid access to historical patient data, provides preventive service recommendations, and allows the online ordering of additional tests. It is designed to work in harmony with the existing hospital EMR by retrieving relevant information from medical records and requiring only minimal additional input from physicians regarding patient-specific risk factors. This facilitates the generation of personalized recommendations for disease prevention and screening.


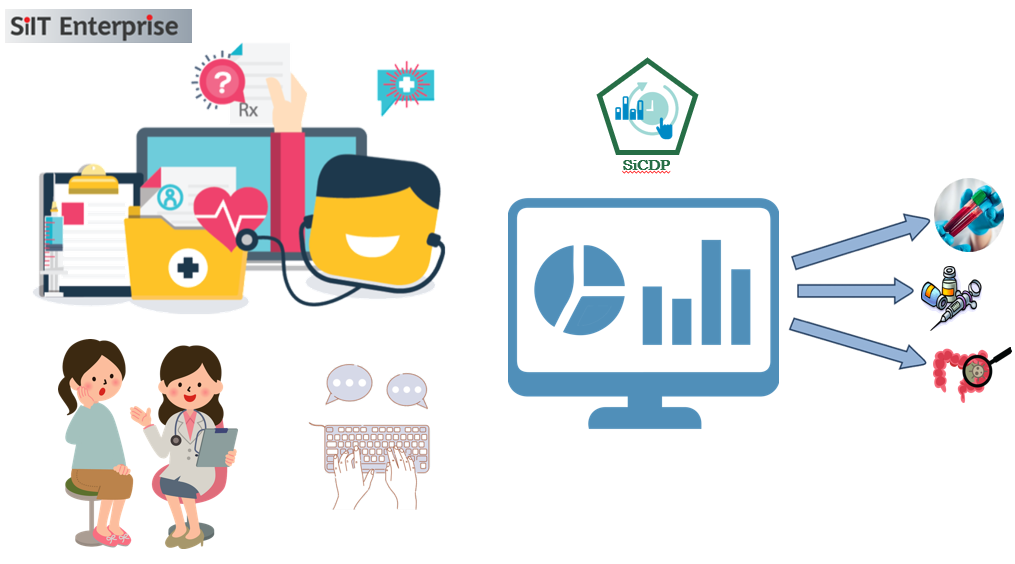


**Figure S2.** A graphic illustrating the functions of the new system

(**Si**riraj **C**linical **D**ecision support system in **P**reventive services, SiCDP)

**Part 3**

Key features of the system include clinical guidance on cancer screening and personalized vaccination, along with access to the most up-to-date clinical guidelines. Physicians are required to provide only a small amount of additional history focused on key risk factors that influence the choice of preventive services.


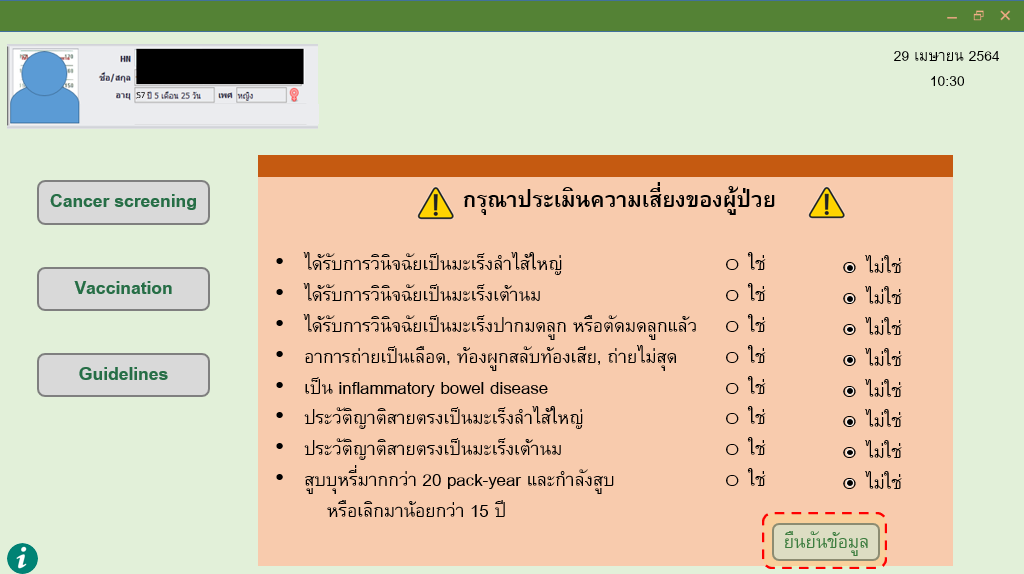


**Figure S3.** Key history-taking points for cancer risk assessment


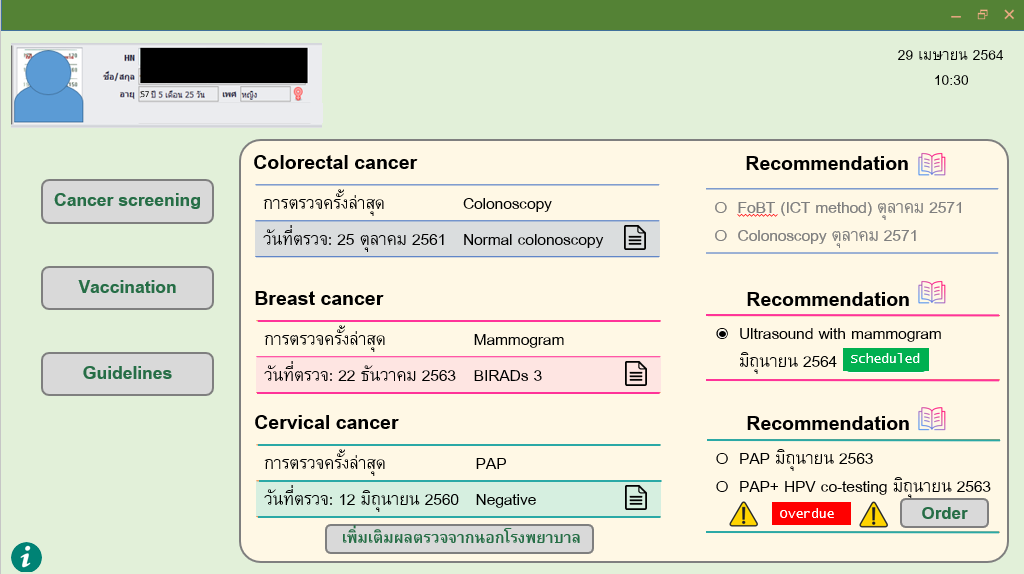


**Figure S4**. A summary of historical cancer screenings and personalized recommendations for further screening

**Part 4**

Additionally, the system’s database is updated every 4 months to remain consistent with the latest clinical guidelines. Users encountering any difficulties can consult the online user manual or seek assistance from the IT Helpdesk.


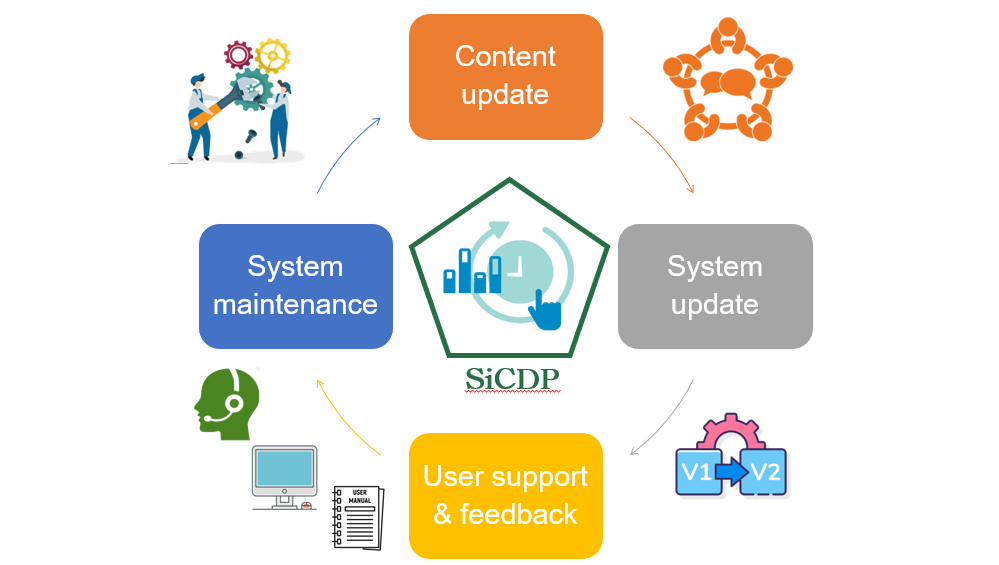


**Figure S5.** A graphic showing the system maintenance and user support features
