# Supplementary material for: Factors Influencing Health Workers’ Acceptance of Guideline-Based Clinical Decision Support Systems for Preventive Services in Thailand: Questionnaire-Based Study
Source: JMIR Hum Factors. 2025 Jul 16;12:e57314. doi: 10.2196/57314 (PMC12286562; doi:10.2196/57314)
Supplement: Multimedia Appendix 2 [file humanfactors-v12-e57314-s002.docx]

| Determinants of intentions and usage | Medical student subgroup | | Graduated physician subgroup | |
| --- | --- | --- | --- | --- |
|  | Crude OR^a^ (95% CI) | *P* value | Crude OR (95% CI) | *P* value |
| Physician’s attitude^b^ | 8.00 (1.48‐43.20)^c^ | .02 | 10.40 (1.93-56.04)^d^ | <.001 |
| Performance expectancy^b^ | 2.68 (0.65‐11.13) | .18 | 3.33 (0.68‐16.32) | .14 |
| Effort expectancy^b^ | 10.50 (1.21‐91.01)^c^ | .03 | 12.67 (1.40-114.41)^c^ | .02 |
| Social influence^b^ | 1.29 (0.31‐5.28) | .73 | 0.68 (0.15‐3.10) | .63 |
| Facilitating conditions^b^ | 1.19 (0.29‐4.95) | .81 | 2.63 (0.58‐11.94) | .21 |

^a^OR: odds ratio.

^b^Where values lower than the mean of the total score were treated as baseline, ie, odds ratio=1.

^c^P<.05.

^d^P<.001.
